# Lyotropic Liquid Crystalline Materials

**DOI:** 10.3390/ma19122485

**Published:** 2026-06-10

**Authors:** Antônio Martins Figueiredo Neto

**Affiliations:** Institute of Physics, University of São Paulo, São Paulo 05508-090, Brazil; afigueiredo@if.usp.br

**Keywords:** lyotropic, structure, phase diagram, phase transition

## Abstract

Liquid crystals are intermediate states of matter, between the isotropic liquid and solid crystal. In this review we will focus on the lyotropic mixtures of materials that present ordering of their basic units and originate remarkable mesomorphic states. We discuss lyotropic materials made of amphiphilic molecules, chromonic molecules, inorganic materials and living systems. We also discuss the relations of lyotropics with biological systems and different applications of lyotropics in the food and cosmetic industry, and in drug delivery. These materials are also used as nanoreactors to produce nanomaterials on this length scale. In summary, lyotropics show a rich set of structures, obtained by their basic units self-assembly, with different symmetries, that allow their application and approach in many branches of science and technology. Moreover, lyotropics still present challenges from the theoretical point of view that are interesting to be studied, for example, the nano segregation occurring in their structure where more than one type of amphiphilic molecule is present in the mixture.

## 1. Introduction

The word “lyotropic” has its roots in Ancient Greek, bringing the idea of *changes* and *dilution*. The term carries the meaning of a new characteristic (i.e., emergence of order) present when a particular solute is dissolved in a solvent or, more properly, a carrier fluid. This fluid may be nonpolar (e.g., an oil) or polar (e.g., water). The first experimental evidence reported in a paper of a lyotropic system dates from the 19th century, by R. Virchow [1]. Aqueous solutions of myelin showed unusual mechanical and optical behaviours, with the presence of thread-like structures in the fluid. Four decades after Virchow’s observations, Lehmann reported an interesting study of an aqueous solution of ammonium oleate [2]. These experiments, performed in a polarising microscope, revealed the formation of amazing textures.

In fact, all these observations and further characterisations of new systems employing different experimental techniques and the development of theoretical models led to the definition of a new state of matter, the *liquid crystalline state* [3,4,5].

The carrier fluid may be electrically polar (e.g., water) or nonpolar (e.g., mineral oil). The dispersed material (solute) in the fluid can be from different types [5]: surfactants, amphiphiles, nanoparticles, biological membranes, graphene oxides, viruses, cellulose, DNA/RNA, polymers, among others. These characteristics make lyotropics remarkable materials not only to investigate fundamental properties of soft matter, but also to develop disruptive technological applications in industry and even in biology and medicine.

The topology of the phase diagrams of lyotropics exhibits structures formed by aggregates of the solute, of different shapes and symmetries. The variety of these structures ranges from micellar (aggregates with small shape anisotropy) to highly anisotropic molecular aggregates and to extensive bicontinuous surfaces. The stabilisation of these phases depends on the temperature, pressure, and relative concentrations of the mixture components [4].

The formation of molecular super-structures in lyotropic liquid crystals is the result of the interaction between the solute molecules (described by the pair potential or potential of mean force), the solvent–solvent molecules, and the solute–solvent molecules [6]. In the case of water molecules employed as the solvent, the presence of the super-molecular aggregates increases the entropy of the whole system and reduces the electrostatic interaction between polar regions of the solute and solvent. This thermodynamic balance leads to the self-segregation phenomenon in lyotropics. From the theoretical point of view, the interaction potential between molecules (e.g., *w*(*r*), *r* being the distance between them) allows the calculation of the force one molecule experiences. This molecule–molecule interaction in the case of solutes in a solvent is affected by the presence and electrostatic properties of the solvent. To go further in the theoretical description of solute molecule self-aggregation, it is necessary to propose models. These are usually ad hoc and adapted to the particular system one wants to describe.

In this review lyotropic mixtures based on amphiphilic molecules, chromonic molecules, inorganic, and biological basic elements will be presented. Moreover, some remarkable applications in different branches of medicine and industry will be discussed. An extensive bibliography is provided. Readers interested in specific aspects of lyotropics may consult the books about the subject, some of them referred in this review.

## 2. Lyotropics with Amphiphilic Molecules

Molecules presenting in their atomic structure regions with different polar and nonpolar characteristics are named amphiphilic molecules. A polar moiety, which may be ionic (e.g., fatty acid salts) or non-ionic (e.g., detergents), forms the hydrophilic part of the molecule. The hydrophobic tail, with one or more non-polar hydrocarbon chains, constitutes the non-polar moiety of the molecule. Common examples include phospholipids, such as those found in biological membranes, and various surfactants.

When amphiphilic molecules are in contact with a solvent, above a critical concentration (critical micellar concentration—cmc), given temperature (T) and pressure (P), the phenomenon of self-assembly occurs, and super-structures may be formed. A mechanism responsible for the self-assembly is the hydrophilic/hydrophobic effect, which is driven by the minimisation of the system’s Gibbs free energy. This effect forces the nonpolar tails to aggregate, sequestering them from the aqueous environment, while the hydrophilic heads form a protective shell shield. Micro-phase segregation occurs on the nanometre scale. The macroscopic symmetry of the resulting phase is dictated by the volume balance between the hydrophilic and hydrophobic portions.

**Figure 1:**
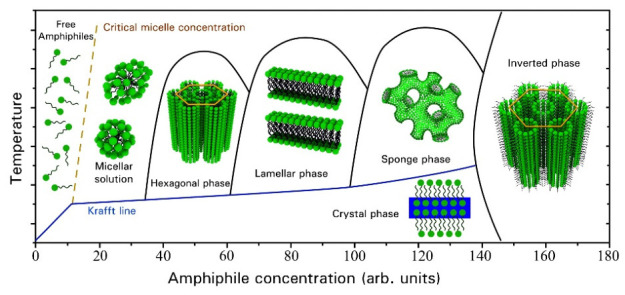
Sketch of a partial phase diagram of a lyotropic mixture of amphiphilic molecules and water. In this representation, mesophase stabilisation depends on the temperature and relative concentration of the solute/solvent. The last structure in the right corner represents a mixture of amphiphile/water/non-polar solvent. The non-polar solvent is located between the cylinders and the water in the centre of them. Inspired by ref. [9].

The polymorphism of the phase diagrams of amphiphilic lyotropics is very rich. At fixed T and P, above the Krafft& line, which separates the hydrated crystalline domain from the liquid crystalline region, different structures may be stabilised (Figure 1). In low concentrations of amphiphilic molecules, no molecular aggregation occurs. Increasing this concentration, micelles of different shapes, such as spheres and less symmetric aggregates (the layer, e.g., in the presence of a co-surfactant), are formed. Remarkably, in this region of the partial phase diagram of a mixture with a surfactant/co-surfactant/water, a fluid with biaxial symmetry was identified [7]. The micelles were modelled as triaxial ellipsoids [4,8] and this complex fluid presents three main indices of refraction and two optical birefringences. By further increasing the concentration of amphiphilic molecules, a hexagonal structure, with long cylinders (~100 nm), and the lamellar mesophase are stabilised. Bicontinuous mesophases (e.g., sponge mesophases) may be stabilised at higher amphiphile concentrations.

In the case of two solvents present in the mixture, one polar and another nonpolar, inverted mesophases can be stabilised. An example is shown in the right corner of Figure 1. A hexagonal inverted structure is depicted where the polar solvent is located in the inner part of the cylinders (in contact with the polar heads of the amphiphile) and the nonpolar solvent is located between cylinders, in contact with the hydrophobic carbonic chains.

**Figure 2:**
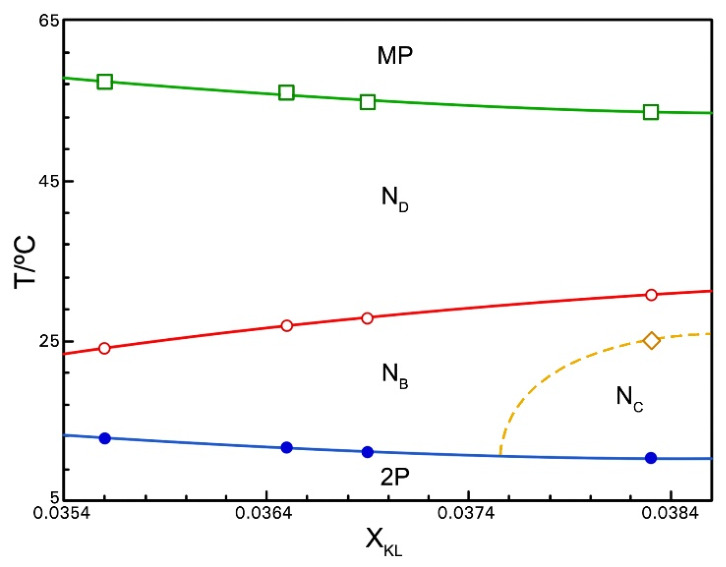
Partial phase diagram of the mixture Potassium Laurate/K_2_SO_4_/UndeOH/H_2_O surface (at XK_2_SO_4_ = 0.0060 and XUndeOH = 0.0114). The solid and dashed lines are used as eye guides. The labels 2P and MP represent a two-phase and a multi-phase region, respectively. X represents molar fraction. Adapted from [10].

The transitions between the different mesophases are usually of first-order [3,4]. On the other hand, the transitions between the uniaxial and biaxial nematic phases are of second order, well described by the Landau mean-field theory. These transitions are driven by modifications of the shape anisotropy of the micelles that trigger their different orientational fluctuations. Lyotropic mixtures that present three nematic mesophases, one biaxial (NB) and two uniaxial (NC and ND—calamitic and discotic, respectively), have a surfactant, a co-surfactant, and the solvent (e.g., water), with or without electrolytes [10]. Figure 2 depicts the partial phase diagram of the mixture Potassium Laurate (KL)/1-undecanol (UndeOH)/K_2_SO_4_/water where three nematic phases are present. It is important to stress that the presence of the co-surfactant in the mixture is essential to stabilise the three nematic phases. Moreover, a pseudo-lamellar structure was experimentally observed in the three nematic phases. The set of experimental evidence allowed the proposal of the Intrinsic Biaxial Micelle model (IBM) to describe the partial phase diagram and transitions between the different nematic mesophases [4]. In the case of lyotropic nematics presenting the three nematic phases, fluctuations are related to small changes in the shape anisotropy of the micelles in the vicinity of the phase transitions. They are due, essentially, to small modifications of the self-aggregation process that stabilise the shape anisotropy of the micelles, driven by the temperature.

**Figure 3:**
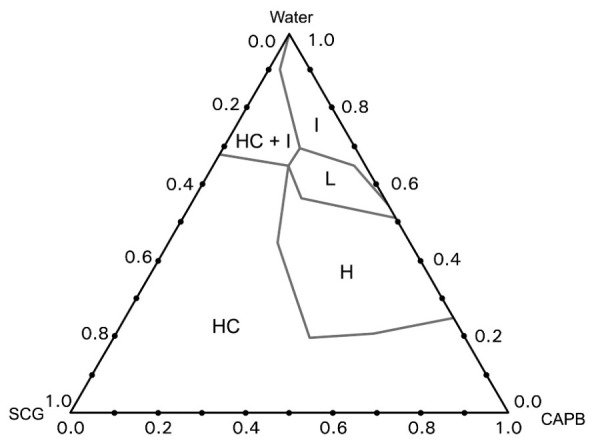
Isothermal phase diagram of the ternary mixture sodium cocoyl glycinate (SCG)/cocamidopropyl betaine (CAPB)/H_2_O. HC, I, L, and H represent the hydrated crystal, micellar isotropic, lamellar, and hexagonal mesophases, respectively. Adapted from [11].

An alternative way to represent the partial phase diagram of a ternary lyotropic mixture is shown in Figure 3, where the temperature is fixed at 25 °C [11].

Biocompatible lyotropic materials have been widely used in the cosmetic industry since their first studies. The property of self-assembly in structures that depend on the relative concentrations of their constituents may deliver or remove substances from the human skin, with benefits in beauty treatments and therapies [12]. Applications of lyotropics in industry and medicine will be discussed in an upcoming section of this review.

Chiral lyotropic liquid crystals may be stabilised by doping the former lyotropic mixture with chiral molecules. Basically, two families of chiral lyotropics were identified, one of them composed of micelles (e.g., molecular aggregates of small shape anisotropy) and another with aggregates of higher shape anisotropy (e.g., lamellae). The doping with chiral molecules can be done by changing the main amphiphilic molecule of the mixture with a chiral version of it or by introducing a new chiral component to the mixture. Chiral lyotropics obtained by the first method are named *intrinsic cholesterics* and by the second, *extrinsic cholesterics*.

**Figure 4:**
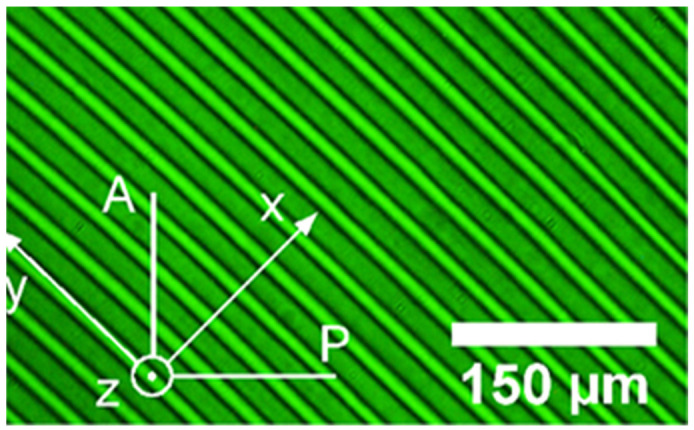
Optical microscopy texture of the mixture Potassium Laurate/1-nonanol/Potassium Sulphate/water/Brucine, in the *Ch*_B_ phase. Temperature of 12.50 °C. The labels P and A refer to the polariser and analyser. Reproduced from [13].

**Figure 5:**
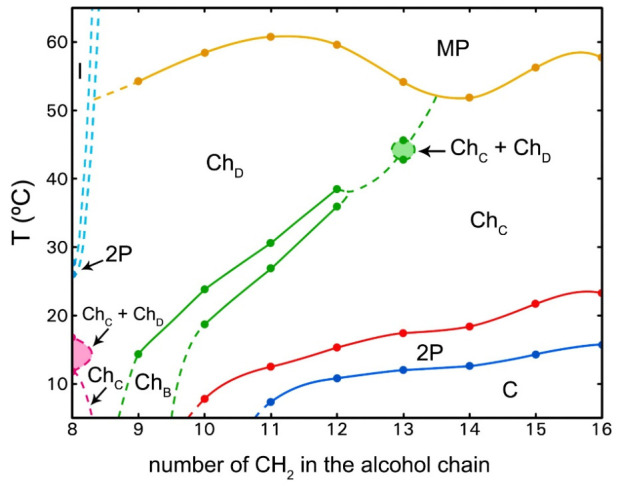
Partial phase diagram of the mixture Potassium Laurate/alcohol/Potassium Sulphate/water/Brucine Lyotropic Mixture, as a function of the number (*n*) of *CH*_2_ in the chain of the alcohol. The labels 2P, MP, I, and C correspond to two phases and multiphase regions, isotropic, and crystalline phase, respectively. The lines are used as an eye guide only. Reproduced from [13].

When lyotropic mixtures presenting the three nematic mesophases are doped with chiral molecules (e.g., Brucine Sulphate, cholesterol, d-alanine, d-tartaric acid, among others) [5], three cholesteric mesophases may be observed: cholesteric calamitic (*Ch*_C_), cholesteric discotic (*Ch*_D_), and cholesteric biaxial (*Ch*_B_). Figure 4 shows the optical microscopy texture of the mixture Potassium Laurate/1-nonanol/Potassium Sulphate/water/Brucine, in the *Ch*_B_ phase, slab sample with the helical axis parallel to the *x* axis of the laboratory frame axis, sample between crossed polarisers (*P* and *A*) [13]. The cholesteric pitch (*P*) was shown to scale with the inverse of the chiral molecule concentration (*c*_m_), i.e., *P*^−1^ = *αc*_m_ [14]. In the limit of *c*_m_ → 0, *P* → ∞, which recovers the nematic characteristic of the mesophase. The constant *α* is named Helical Twisting Power and depends on the temperature, components of the mixture, and their relative concentrations. Figure 5 shows the partial phase diagram of the mixture Potassium Laurate/alcohol/Potassium Sulphate/water/Brucine Lyotropic Mixture, as a function of the number (*n*) of *CH*_2_ in the chain of the alcohol [13].

From the theoretical point of view, the doping of the former lyotropic mixture with chiral molecules may be modelled as an *applied chirality field* [15,16,17]. It has been experimentally shown that the chirality field renders the *Ch*_D_ to *Ch*_B_ transition continuous, differently from what was observed in the nematic mesophases.

**Figure 6:**
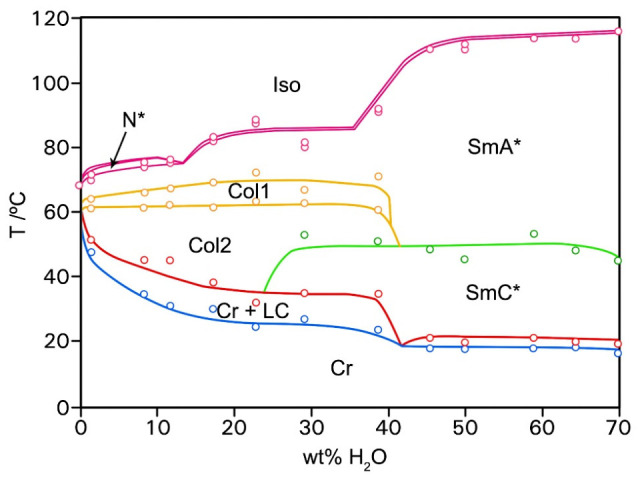
Phase diagram of the mixture diol 1/water. The labels Iso, N*, Col, Cr, LC, SmA*, SmC* represent isotropic, chiral nematic, columnar, crystal, liquid crystal, chiral smectic A, and chiral smectic C, respectively. Reproduced from [18].

The chiral characteristic was observed in smectic-type mesophases from mixtures of diol 1/water (or formamide) [18]. A chiral smectic C structure (SmC*) was identified by different experimental techniques. The diol 1 molecule combines lyotropic and thermotropic behaviours, favouring the in-layer molecule-to-molecule interaction, which favours the smectic-type structure, and the chiral characteristic favours the interlayer interaction stabilising a structure equivalent to the thermotropic SmC*. Moreover, a chiral smectic A (SmA*) mesophase was also identified in the phase diagram of this mixture, revealing again the rich polymorphism of lyotropics. Figure 6 shows the phase diagram of the mixture diol 1/water, where the SmC* was identified. A remarkable characteristic of the SmC* is the spontaneous polarisation and the surface-stabilised states under the action of an external electric field. Two monotropic columnar mesophases (Col1 and Col2) were also observed in the phase diagram (Figure 6).

A clever procedure involving lyotropic mixtures and a gel led to the formation of a complex material with properties from both systems: the ordering from the liquid crystal and the soft rigidity of the gel. This process is the gelation of a lyotropic mixture, where the soft solids obtained incorporate the lamellar liquid crystalline structure [19].

More recently, interesting research was carried out to demonstrate the self-assembly of amphiphilic molecules in protic ionic liquids, stabilising liquid crystalline structures [20]. Monoolein/protic ionic liquid mixtures were prepared and the liquid crystalline mesophases stabilised were the lamellar, inverse hexagonal, gyroid cubic, and diamond cubic. The experimental techniques employed in the identification of the different mesophases were the usual ones: the polarised optical microscope to observe the textures, and the SAXS to determine the nanoscopic structure.

## 3. Lyotropics with Chromonic Molecules

Chromonic molecules are aromatic molecules, where the atoms and rings are distributed in a flat structure. Ionic domains are located on the periphery of the molecule. Interestingly, aqueous solutions of this type of molecule lead to the stabilisation of liquid crystalline mesophases [5].

The presence of the ionic groups in the molecules is essential to promote good solubility in polar solvents. Since the core of the chromonic molecules is hydrophobic and the periphery is hydrophilic, in the presence of water, the molecules pile up in columns, whose length depends on the solute concentration. *π*-stacking interaction between neighbouring molecules favours the stabilisation of the columns. Water molecules are removed from the face-to-face molecular structure, occupying the space between columns. Differently from the self-aggregation process occurring in amphiphilic lyotropics, in chromonics no critical micelle concentration is observed. Even at a low molecular concentration (e.g., isotropic phase), small columnar aggregates exist in the mixture.

Examples of chromonic molecules showing lyotropic mesophases are disodium cromoglycate (DSCG), methyl orange, Sirius supra brown RLL, and Sunset Yellow. Some more complex mixtures stabilise chromonic mesophases such as, for example, adietic acid/polyethylene glycol/water [21].

**Figure 7:**
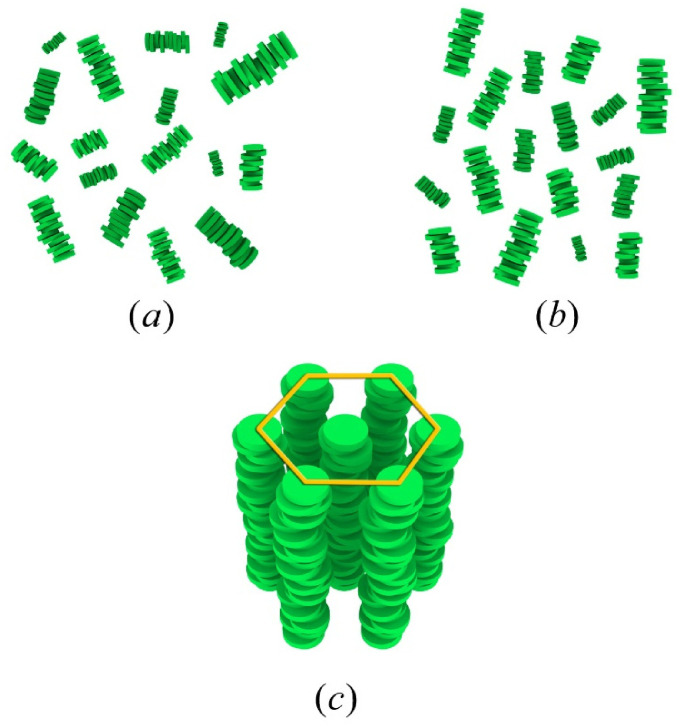
Sketches of the chromonic mesophases. Discoid molecules pile up in cylinders of different lengths. (**a**) Isotropic; (**b**) uniaxial nematic phase; (**c**) hexagonal phase.

**Figure 8:**
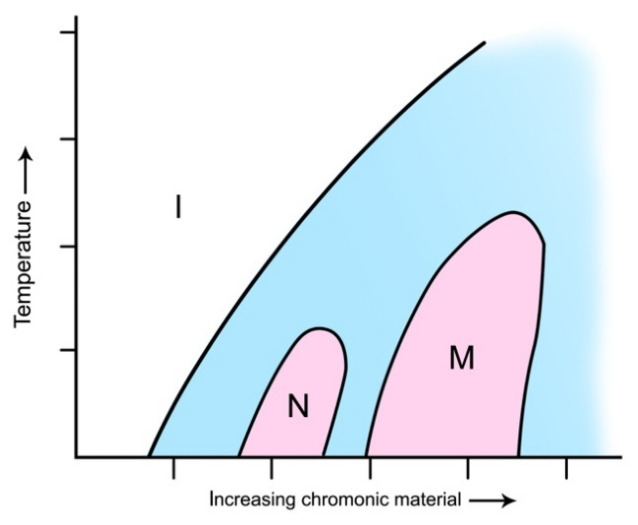
Sketch of a typical phase diagram of a chromonic molecule/water mixture. I, N and M represent the isotropic, nematic and hexagonal mesophases. The blue light represents phase coexistence regions, typical of a first-order transition.

The mesophases observed in lyotropic chromonics are isotropic (I), uniaxial calamitic nematic (N), and hexagonal (M) (Figure 7). The transition from nematic to hexagonal was shown to be of first-order, as imposed by the symmetries of these phases. However, the transition may be characterised as weakly first-order, and a coexistence domain is experimentally observed in the frontier of the N and M mesophases. A sketch of a typical phase diagram of a chromonic molecule/water mixture is depicted in Figure 8.

Differently from the usual thermotropic materials, the property of modifying the length of the stack as a function of the relative molecular concentration and temperature directly affects the elastic properties of chromonics. Normally, the order of magnitude of the three Frank elastic constants of splay (*K*_11_), twist (*K*_22_), and bend (*K*_33_) [22] is the same. In the case of chromonic nematics, *K*_22_ is about one order of magnitude smaller than the other constants [23,24]. The viscosities (*η*_splay_, *η*_twist_, *η*_bend_), also measured in the chromonic nematic phase, revealed that *η*_splay,twist_ are about four orders of magnitude higher than that of *η*_bend_ [25]. This result may be due to the presence of long molecular stacks in the nematic mesophase, which render splay and bend deformations more difficult. However, this is still an issue of discussion, and more experiments with different chromonic molecules are needed.

An interesting field of research yet underexplored by experimentalists and theoreticians is the doping of chromonic lyotropics with chiral molecules. Fundamental questions about the stabilisation of a long-range cholesteric ordering of the molecular stacks in a chromonic nematic doped with chiral molecules are still unanswered. This aspect brings new challenges from the theoretical point of view, since the value of the splay and twist elastic constant in chromonics is so big, and very different from the bend elastic constant and from those encountered in thermotropic liquid crystals.

In the two-phase (isotropic and nematic) coexistence region of the phase diagram, nematic droplets can be stabilised in the isotropic medium. The texture of these droplets under a polarising optical microscope was investigated as a function of an external magnetic field, showing twisted bipolar patterns [26]. The results show the role of the boundary conditions in the dynamic behaviour of the structure in these confined conditions.

The Lehmann effect, where these droplets spontaneously rotate in the presence of a heat flux, essentially driven by a temperature gradient, was also observed in this biphasic domain of chromonic liquid crystals [27]. The rotational speed velocity of the droplet was shown to be proportional to the temperature gradient and inversely proportional to its radius.

## 4. Lyotropics with Inorganic Materials

Aqueous mixtures of vanadium pentoxide (V_2_O_5_) needle-like particles were shown to stabilise lyotropic liquid crystalline mesophases [28]. These particles are big, compared with the basic units of thermotropics and lyotropics previously discussed here. The needles show lengths of the order of the micron, large shape anisotropy, with diameters smaller than 0.1 μm. Mixtures of these needles in water, after vigorous shaking, form birefringent regions, named tactoids, in an isotropic matrix. From the theoretical point of view, approaches based on Onsager’s model describe the stabilisation of a nematic ordering in those birefringent domains [29]. Since the basic units (needles) in the mixture are big, ageing effects were observed in the system, becoming a drawback in the definition of a (true) liquid crystalline structure, which is expected to be thermodynamically stable.

Other materials suspended in water also formed similar lyotropic nematic phases, such as aluminium oxyhydroxide [30], iron oxyhydroxide [31], imogolite [32], graphene oxide [33], lithium molybdenoselenite [34]. Some of the aqueous suspensions of these materials form liquid crystalline mesophases like the nematic and smectic.

Plate-like mineral particles, of typical in-plane dimension in the range from 3 μm to about 30 μm and thickness of the order of nanometres, dispersed in water, also show birefringence, indicating a liquid crystalline-like behaviour. Water molecules are mainly located between the plates. Nematic-like structures were identified in these suspensions.

**Figure 9:**
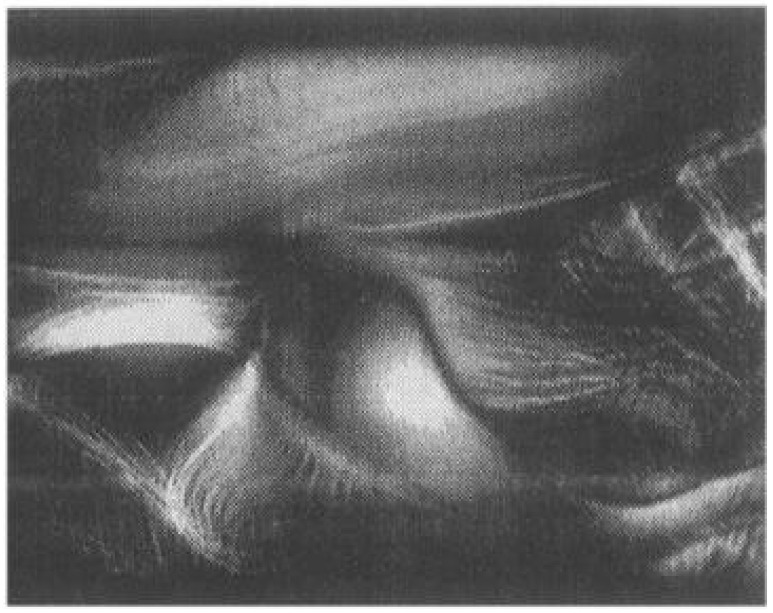
Bentonite plates/water mixture observed in a polarised optical microscope. Augmentation 50×. Birefringent nematic gel mesophase. Reproduced with permission from [35].

Examples are montmorillonites [35] and gibbsite [36]. Figure 9 shows a texture in a polarised optical microscope of the mixture bentonite plates/water, in the birefringent nematic gel phase.

## 5. Lyotropics and Biology

This topic, besides its interest from a fundamental point of view, allowed remarkable applications of lyotropics in medicine as a drug carrier to treat different types of pathologies.

There are some living systems whose basic units are viruses dispersed in a bio-compatible fluid. An advantage of this type of system is that all the basic units have the same shape and dimension, which allows an easier phenomenological description.

An example of this system is the Tobacco Mosaic Virus (TMV) suspended in water/borate fluid [37]. These viruses are cylindrical, with a typical length of about 300 nm and a diameter of about 30 nm. At virus concentrations around 0.15 g/cm^3^, at room temperature, TMV suspensions were subjected to an external magnetic field (typically 2 T), and a uniaxial nematic phase was observed and magnetically oriented. The order parameter measured was very high, ~0.9. Decreasing the virus concentration to about 0.10 g/cm^3^, the isotropic phase is stabilised. The coexistence region present between these two phases supports the first-order type phase transition, as expected. Besides the nematic phase, a smectic A was observed at virus concentrations around 180 mg/mL [38]. The nematic to smectic A transition was shown to be of second order, as theoretically predicted and expected from the symmetries involved.

**Figure 10:**
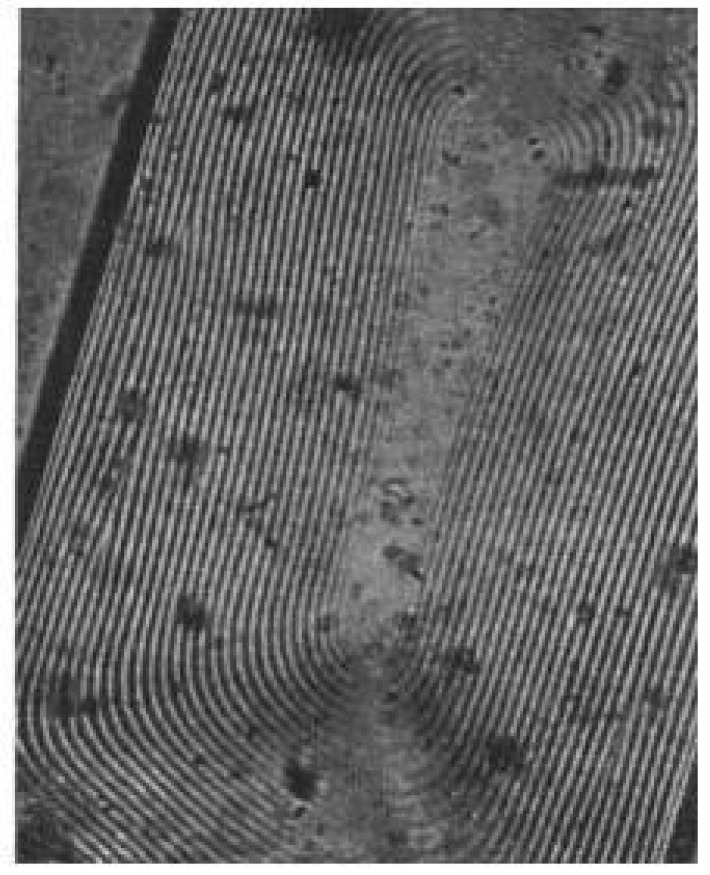
Aqueous suspensions of filamentous phage virus (fd) showing a cholesteric texture, even without crossed polarisers. Concentrated in sealed quartz capillaries. Magnification 60 times. Reproduced with permission from [39].

Filamentous phage virus (fd) is a chiral cylindrical polyelectrolyte ~700 nm long and with a diameter of ~5 nm. Aqueous suspensions of this virus stabilise cholesteric mesophases with typical pitches of about tens of microns [39]. As observed in thermotropics and amphiphilic lyotropics, the pitch of the cholesteric structure decreases with the increasing concentration of the chiral component, in this case, the virus. Figure 10 shows a cholesteric texture in a suspension of filamentous phage virus (fd) in water, observed in an optical microscope.

**Figure 11:**
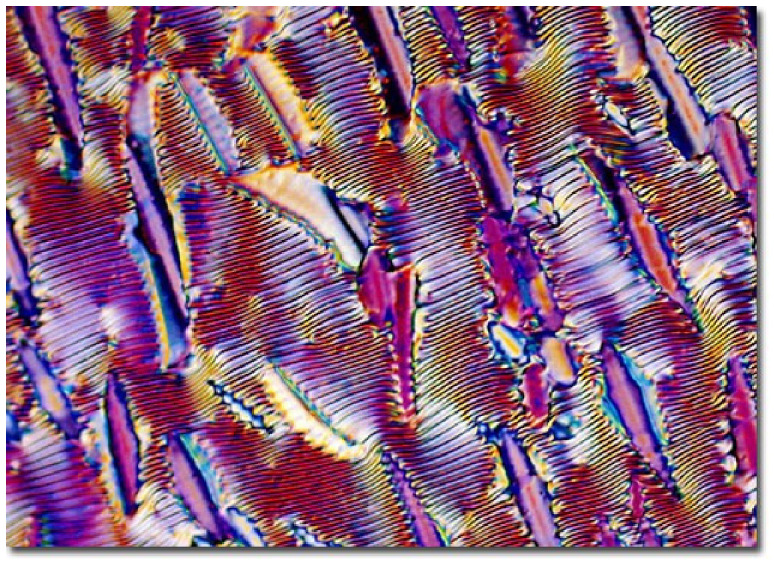
Texture with batonnets in magnetically aligned (5 T) aqueous DNA solutions. Magnification of about 600×, microscope with crossed polarised illumination. Reproduced with permission from Molecular Expressions and Florida State University https://micro.magnet.fsu.edu/dna/pages/magneticfield4.html (accessed on 21 April 2026).

DNA and RNA macromolecules may also show lyotropic behaviour when their lengths are of the order of tens of nanometres. Suspensions of these types of basic units in water, with the addition of ions, stabilise liquid crystalline structures as a function of the temperature and concentration of the units. The typical sequence of mesophases observed in these suspensions, as a function of increasing concentrations of the units, is isotropic—blue phase—cholesteric—columnar hexagonal. By increasing the units’ concentration further, the crystalline phase is achieved [5,40]. Figure 11 shows an optical microscopic texture (crossed polarisers) of an aqueous DNA solution, with batonnets, magnetically aligned in a 5 T field. The typical finger-print texture is present in the texture. Interestingly, these suspensions show the blue phase in the region between the isotropic and the cholesteric mesophase.

Suspensions of cellulose nanocrystals in water also show lyotropic liquid crystalline mesophases. As observed in amphiphilic lyotropics, a critical concentration of nanocrystals is needed to stabilise lyotropic structures [41]. These crystals are rod-shaped, with lengths of the order of 150 nm and widths of ~5 nm. Typically, at nanocrystal concentrations of about 5.5 × 10^−6^ nm^−3^, the cholesteric mesophase is stabilised. At lower concentrations, only the isotropic phase is present in the phase diagram. Not only does pure cellulose form the basic units of lyotropics, but also its derivatives, obtained from chemical reactions such as, for example, esterification, etherification, and oxidation. Suspensions of these materials stabilised not only cholesteric mesophases, but also uniaxial nematic mesophases [42].

**Figure 12:**
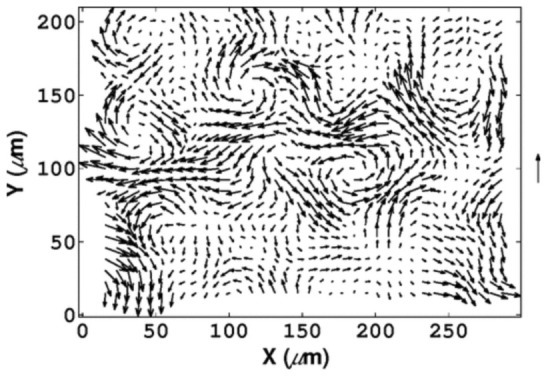
Vector field of the 1085 and YB886 of *Bacillus subtilis* bacteria in solution. The arrows indicate the direction of the movement and the bacteria’s velocity. The arrow at right represents the velocity of 35 μm/s. Observation in the XY plane and the scales are in μm. Reproduced with permission from [43].

The richness of lyotropic systems is not limited to the system of molecular aggregates, macromolecules, inorganic particles, and viruses. In the beginning of the 21st century, liquid crystalline-like structures were observed in systems of bacteria (in this case, the basic unit) dispersed in biocompatible fluids [43]. An example is the suspensions of strains 1085 and YB886 of *Bacillus subtilis*. This is a peritrichously flagellated rod-shaped bacterium with a length of ~4 μm and a diameter of ~0.7 μm. This type of system was named “living liquid crystals” [44]. At high concentrations of bacteria, collective large-scale movements were observed that resemble the texture observed in liquid crystalline materials. Figure 12 shows the vector field of the velocities of *Bacillus subtilis* in suspension. The length of the arrows indicates the value of the bacteria velocity, calibrated by the arrow on the right side of the figure. A biocompatible fluid where bacteria can be suspended is an aqueous suspension of chromonic molecules in the uniaxial nematic phase [45]. The topology of the nematic director paves the way for bacteria movement. Disclinations, typical of unaligned nematics in slabs, were observed and the dynamics of the texture could be followed with the bacteria decoration.

## 6. Applications of Lyotropics

Traditional applications of lyotropics can be encountered in the food and cosmetics industries, involving different aspects of products. Their uses focus on consistent sensation, aroma, conservation, among others [5,46].

Aqueous-based lyotropic mixtures certainly are those with more evident biological applications. However, non-aqueous systems also show interesting and very useful applications [47].

One of the most remarkable and interesting applications of lyotropics is drug delivery. The richness of structures, biocompatibility, and composition (i.e., of their basic units) gives these systems a versatility not present in other materials. In particular, amphiphilic lyotropics may incorporate in their structure active molecules with both hydrophobic and hydrophilic behaviour, depending on the mixture composition. Since the stabilisation of a liquid crystalline structure depends on different factors, such as, for example, temperature, pH of the medium, and presence of surrounding molecules, a structural phase transition may occur and the active molecule incorporated in the lyotropic structure may be delivered (e.g., glucose, paclitaxel, diazepam, irinotecan, among others) [48].

Drug delivery based on lyotropics employs different mechanisms, not only the gastrointestinal and blood circulatory systems, but also transdermal delivery has been tested with relative success. The latter, in particular, is used to deliver lipophilic drugs [49,50]. It has been shown that the incorporation of active drugs (e.g., nifedipine), at least in the concentration investigated, did not change the original structure of the mesophase. The efficiency of the delivery depends on the particular mesophase structure (e.g., lamellar, cubic and hexagonal) and is triggered according to the patient’s needs.

**Figure 13:**
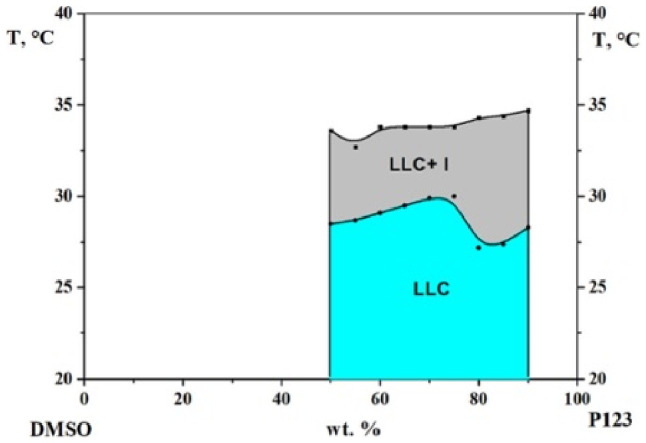
Phase diagram of the Pluronic P123 (PEO20PPO70PEO20)/dimethyl sulfoxide (DMSO) lyotropic mixture. Regions of the lamellar mesophase and of the lamellar + isotropic coexistence phases. Reproduced with permission from [51].

The use of transdermal delivery has to pass through the stratum corneum of the skin. Biocompatible formulations of lyotropics are strong candidates to overcome this barrier and deliver drugs through the skin in controlled doses. Lyotropic mixtures of Pluronic P123 (PEO20PPO70PEO20)/dimethyl sulfoxide (DMSO)/ibuprofen were prepared and their efficiency as a transdermal drug delivery vector was evaluated in terms of rheological and adhesive properties [51]. The lyotropic structures encountered in these systems were the lamellar and isotropic (Figure 13). An aspect that should be investigated in more detail in further experiments is the orientation of the lamellar structure with respect to the skin. Depending on the particular hydrophobic or hydrophilic drug employed to be delivered, and its location in the lamellar structure, the orientation of the lamellae may be an important factor to be considered in the delivery rate.

**Figure 14:**
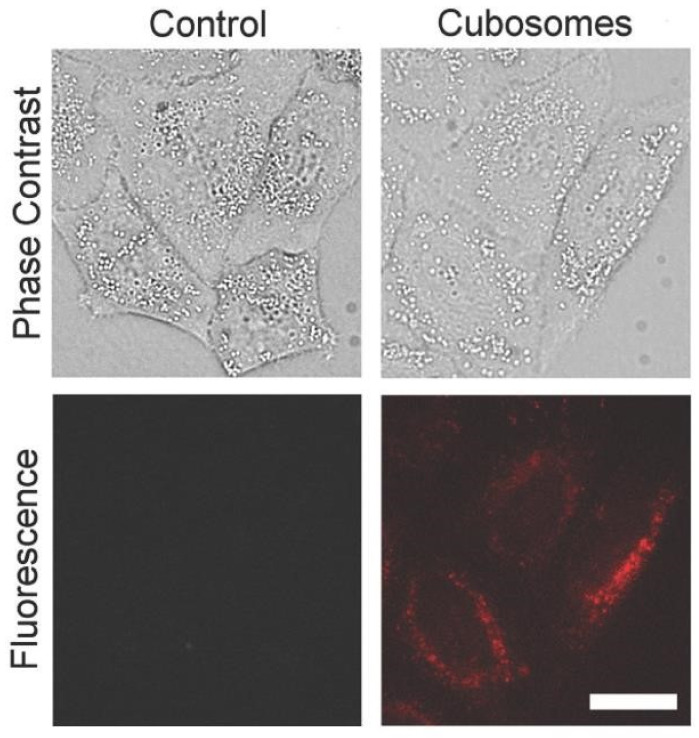
Fluorescence microscopy of living HeLa cells untreated (control) and treated with cubosomes from the lyotropic system docetaxel-loaded nanoparticles stabilised in water by a mixture of pluronic (poly(ethylene oxide)-poly(propylene oxide)-poly(ethylene oxide) triblock copolymer) F108 (PF108) and rhodamine- and folate-conjugated PF108. The scale bar represents 20 μm. Reproduced with permission from [52].

Lyotropic systems are also employed as carriers of theranostic agents [50,52]. An example is the lyotropic system docetaxel-loaded nanoparticles, stabilised in water by a mixture of pluronic (poly(ethylene oxide)-poly(propylene oxide)-poly(ethylene oxide) triblock copolymer) F108 (PF108) and rhodamine- and folate-conjugated PF108, which form cubosome structures [52]. Figure 14 shows images of fluorescence microscopy of living HeLa cells untreated (control) and treated with cubosomes, revealing their uptake.

Lyotropic liquid crystalline systems are used in the treatment of glaucoma, essentially employing biofilm gels in the cubic and hexagonal structures, in the form of eye drops [53]. Other pathologies may be treated by using lyotropic formulations through the nasal cavity. The idea is to deliver drugs through the nose mucosa, a highly vascularised region, allowing quick drug absorption and delivery to the blood. One of the more remarkable illnesses treated with this method is Parkinson’s disease [54]. The lyotropic mixture employed in this experiment is the glyceryl monooleate/N-methylpyrrolidone/water, and the drug *levodopa*. The SAXS characterisation of the liquid crystalline mesophase revealed a cubic structure. The absorption of the drug by the nasal mucosa has shown the brain bioavailability of the method, at least in the animal test performed.

Certainly, more advances are expected with the progress coming from the numerous results from interdisciplinary groups working to improve drug delivery in humans [55]. The biocompatibility of lyotropics is an outstanding property that allows their use for this purpose. Teams putting together physicists, chemists, immunologists, biologists, and physicians join expertise from different branches of science, allowing creative solutions for actual challenges not yet achieved.

The richness of structures of lyotropics, varying from bicontinuous to isolated basic units, associated with the hydrophobic/hydrophilic regions present in the same structure, makes them excellent templates to build nanodevices for different applications. Moreover, lyotropic structures present very high internal interfacial surface area. An example is the mixing of a polymer in the original lyotropic system that, depending on its physical–chemical characteristics, will be distributed in the lyotropic structure. After this “filling” process, the entire system is subjected to polymerisation and posterior removal of the components of the lyotropic original mixture. What remains is the polymer structure, reproducing the original lyotropic arrangement [56].

**Figure 15:**
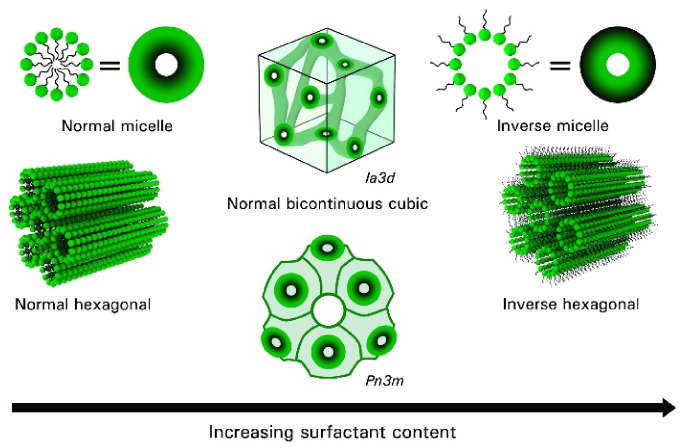
Sketch of the different lyotropic structures used as templates for fluid filters. Ia3d and Pn3m refer to the symmetries of the bicontinuous mesophases. Reproduced with permission from [57].

Nano-filter membranes based on this technology have been used to purify water [57]. One of the advantages of this type of device is its selectivity and high permeability. Figure 15 shows sketches of lyotropic structures used as templates to build active layers for fluid filtering devices.

Since lyotropic mesophase structures separate regions of different electric polarity in the nanoscale, they were used as nanoreactors to produce nanomaterials in this length scale [58]. The idea is simple and clever: confine reagent substances inside the structure of lyotropic mesophases. Metallic and semiconducting nanoparticles were synthesised using this strategy, allowing good control of the particles’ size, with small polydispersity.

An interesting application of lyotropics is as a matrix for 3D crystallisation of protein membranes [59]. It has been shown that the quality of the crystalline structure of crystals obtained with this procedure is very good and the amount is enough to be investigated by usual diffraction and scattering X-ray techniques. This last aspect (small amounts produced) could be a drawback of the procedure using lyotropics; however, it was shown not to be an actual limitation. Obviously, there are issues to be addressed in the use of this procedure concerning the composition of the lyotropic mixture and its compatibility with the crystal one needs to grow. However, the availability of different compositions of the mixtures and the richness of the structures available open up the possibility of design compatible systems to overcome any issues that may arise in the use of this procedure [60].

SnZe nanowires (typical diameter of about 10 nm) were synthesised by using the hexagonal structure of a lyotropic mixture as a template. The mixture is poly(ethylene oxide)–poly(propylene oxide)–poly(ethylene oxide) amphiphilic block copolymer as the surfactant, heptane as the non-polar dispersed phase, and formamide as the polar continuous phase [61].

**Figure 16:**
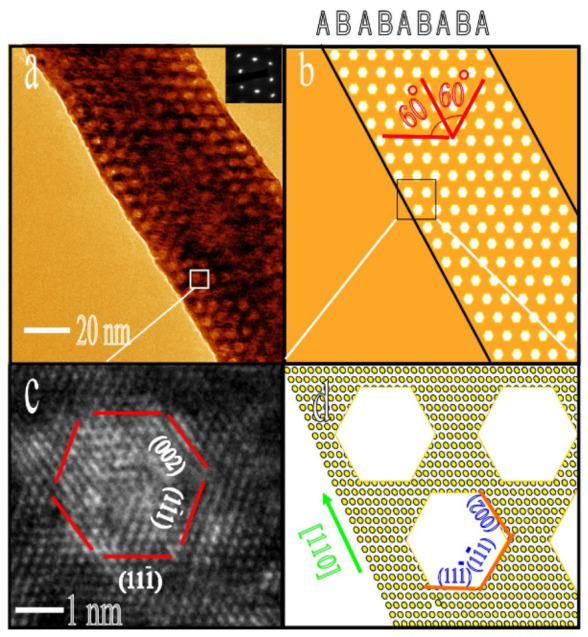
Porous Pt nanowire: (**a**) Transmission electron microscopy (TEM) image of the nanowire and the corresponding electron diffraction patterns (insets). (**b**) Reconstructed image of (**a**). (**c**) High-resolution TEM of a pore. (**d**) Sketch of the crystal structure represented in (**c**). A-B-A-B refers to the mesopores arrangement packing. Reproduced with permission from [62].

Nanostructured metals with highly ordered mesoporosity were synthesised by using lyotropic hexagonal and lamellar mesophases from the mixture of octaethylene glycol monohexadecyl ether (C_16_EO_8_)/H_2_O [62]. The structure of the Pt mesopore is two-dimensionally hexagonally ordered (Figure 16). This procedure allows the preparation of long-range (in the mesoscopic scale) highly ordered structures, which opens up the possibility of new technological applications not yet envisaged.

Chiral nanostructures can also be produced by using lyotropics based on cellulose nanocrystals. The procedure involves the dilution of gold nanorods in the lyotropic mixture and then removing the water [63]. The Au nanorods follow the chiral structure of the cholesteric mesophase and, after the water removal, they keep the cholesteric arrangement. This method allows the fabrication of a chiral plasmonic nanostructure. Since the characteristic of the chiral structure in lyotropics depends on different parameters (e.g., relative concentration of the mixture components, temperature, and pressure), the technique can be adapted to the particular use of the final product.

## 7. Final Remarks

Despite the scientific advances in the understanding of the physical–chemistry present in lyotropic systems, there are some questions not yet completely resolved. This aspect still remains a challenges to researchers, and keeps the subject interesting from the theoretical point of view. One of these questions is the nano segregation of different molecules in systems with more than one amphiphile in the mixture. An example is the stabilisation of orthorhombic micelles that constitute the basic units of lyotropic uniaxial and biaxial nematics in the same phase diagram. The nano segregation of molecules in the micellar scale seems to be one of the causes of this low symmetry micelle formed.

An interesting property of lyotropic systems, one that puts them at the frontier of biological and technological applications, is the dynamics of the molecules that form the molecular super-structures. It is known that the molecules from these aggregates and those (free) in the bulk of the mixture exchange their location on timescales of the order of 10^−5^–10^−3^ s [64]. These dynamics were modelled using the *Collective Small Displacements* model to predict activation energies and transfer timescales [65].

The future of lyotropics as drug delivery systems is still far from being completely explored. New possibilities have been recently proposed to mimic human low-density lipoprotein (LDL) to be taken up by tumours and deliver anti-cancer drugs and metallic nanoparticles.

New and disruptive applications of lyotropic as nanoreactors to promote chemical reactions in small and confined environments are appearing, revealing that reliable processes and technological availability are being developed.

## Data Availability

No new data were created or analysed in this study. Data sharing is not applicable to this article.
